# Dietary supplementation habits of young Canadian athletes

**DOI:** 10.1186/1550-2783-8-S1-P22

**Published:** 2011-11-07

**Authors:** Megan Stadnyk, Jill A Parnell

**Affiliations:** 1 Department of Physical Education and Recreation Studies. Mount Royal University, Calgary, Alberta, Canada, T3E 1W9

## Background

High-performance, adult athletes consume dietary supplements to increase energy, maintain health or prevent nutritional deficiencies and improve exercise recovery; however, dietary supplementation patterns of young, Canadian athletes remain undetermined. Purpose: 1) Determine the types and frequency of dietary supplement use in young athletes. 2) Determine preferred means of educational media for this demographic.

## Methods

A content validated, reliability tested questionnaire was developed to assess dietary supplement use, motivation for supplementation, and preferred means of education. 136 male and 247 female athletes (11-25 years) completed the questionnaire on site by recall.

## Results

93% of athletes report taking some form of dietary supplement with multivitamins, vitamin C, calcium, and sport drinks as the most frequent daily occurrences (30.5%, 29.2%, 27.6% and 19.8% respectively). 18.8% report ingesting energy drinks within the month. The top three reasons for supplement use include: stay healthy 81.0%, increase energy 56.5%, and enhance immune system 52.6%. Family and friends are the primary source of information; however, their preferred means of education were individual consultation, presentations, and the internet.

## Conclusion

Dietary supplement use is common in young athletes. They would prefer to be educated by professionals in individual consultations and presentations; however, they are relying primarily on friends and families. There is a high use of dietary supplements in this demographic yet they lack reliable information. It is essential to develop nutrition education programs for young athletes and to identify the risks and benefits of supplement use in this population.

